# Is There a Relationship Between Aortic Valve Annular Plane Systolic Excursion and Left Ventricular Rotational Mechanics in Healthy Adults? Insights from the MAGYAR-Healthy Study

**DOI:** 10.3390/jcm15166177

**Published:** 2026-08-10

**Authors:** Attila Nemes, Barbara Bordács, Nóra Ambrus, Csaba Lengyel

**Affiliations:** Department of Medicine, Albert Szent-Györgyi Medical School, University of Szeged, Semmelweis Street 8, P.O. Box 427, 6725 Szeged, Hungary; bordacs.barbara.aniko@med.u-szeged.hu (B.B.); ambrusnora@gmail.com (N.A.); lengyel.csaba@med.u-szeged.hu (C.L.)

**Keywords:** three-dimensional, speckle-tracking, echocardiography, aortic valve, annulus, displacement, healthy, association, left ventricular, rotation

## Abstract

**Background/Objectives:** During the cardiac cycle, the left ventricle (LV) exhibits not only a complex contraction–relaxation pattern, but also a wringing-like motion known as LV twist or rotational mechanics. Concurrently, the aortic valve and its annulus (AVA), representing the LV outlet, undergo spatial displacement throughout the cycle, which can be quantified as AVA plane systolic excursion (AAPSE). While three-dimensional speckle-tracking echocardiography (3DSTE) enables the simultaneous assessment of these functional parameters, the relationship between AAPSE and basal/apical LV rotation—particularly across different ranges of these values—remains poorly characterized in healthy subjects. Therefore, the present study aimed to characterize this relationship in detail. **Methods:** This study included 110 apparently healthy adult volunteers (mean age: 35.1 ± 11.9 years; 68 males). All subjects underwent two-dimensional Doppler echocardiography and 3DSTE, with the latter enabling simultaneous quantification of LV rotational parameters and AAPSE. **Results:** A tendency toward increased basal LV rotation and decreased apical LV rotation was observed in healthy subjects with mean AAPSE, while LV twist remained preserved compared to those with lower- or higher-than-mean AAPSE. AAPSE tended to be higher in healthy subjects with mean basal LV rotation compared with those with lower- or higher-than-mean values. Furthermore, AAPSE was significantly increased in subjects with elevated apical LV rotation. AAPSE did not correlate with any LV rotational parameters, including basal LV rotation (r = −0.008, *p* = 0.932), apical LV rotation (r = 0.065, *p* = 0.593), LV twist (r = 0.061, *p* = 0.632), and LV twist time (r = 0.181, *p* = 0.073). **Conclusions:** There are no significant correlations between AAPSE and LV rotational parameters, but the trends observed in subgroup analyses could serve as hypothesis-generating observations for future studies.

## 1. Introduction

The substantial technical advances in echocardiography have made it possible to perform detailed (patho)physiological analyses, even in healthy individuals, which were previously not feasible using non-invasive and easy-to-learn methods. Three-dimensional (3D) speckle-tracking echocardiography (STE), one of the most advanced imaging techniques, uses digital 3D datasets acquired at a given time, enabling the simultaneous assessment of multiple heart chambers and valves. This method allows a comprehensive evaluation of their interactions and mutual functional relationships [1,2,3,4,5]. Such an approach facilitates a deeper understanding of the more complex relationships of the cardiac mechanics.

It is well established that during the cardiac cycle, the left ventricle (LV) not only performs a contraction–relaxation pattern characterized by strains named LV deformation [6], but also a wringing motion resulting from the opposing orientation of epicardial and endocardial muscle fibers perpendicular to each other, referred to as LV twist or LV rotational mechanics. Accordingly, the LV base rotates clockwise, while the LV apex rotates counterclockwise [7,8,9,10,11]. In addition, the aortic valve (AV), considered the outlet of the LV [12], and its annulus (AVA) undergo dynamic spatial displacement during the cardiac cycle, which can be quantified by AVA plane systolic excursion (AAPSE) [13,14]. All these functional parameters can be assessed simultaneously using 3DSTE [15,16,17]. However, the relationship between basal and apical LV rotations and AAPSE remains insufficiently characterized in healthy subjects across different ranges of these parameters. The aim of the present study was to analyze these relationships in detail.

## 2. Subjects and Methods

### 2.1. Subject Population

This exploratory study enrolled 110 apparently healthy adult volunteers (mean age: 35.1 ± 11.9 years; 68 males) between 2011 and 2017. All participants had clinical parameters within normal reference ranges at initial screening, which included physical examination, standard 12-lead electrocardiography (ECG), routine laboratory tests, two-dimensional (2D) echocardiography with Doppler evaluation and 3DSTE. Exclusion criteria ensured the absence of any known disorders or pathological conditions that might affect the findings. Specifically, none of the participants were taking regular medication, were smokers, were pregnant, were athletes, or were obese (defined as body mass index > 30 kg/m^2^). This research is part of the ongoing ‘Motion Analysis of the heart and Great vessels bY three-dimensionAl speckle-tRacking echocardiography in Healthy subjects’ (MAGYAR-Healthy Study). The MAGYAR-Healthy Study focuses on performing physiological analyses between 3DSTE-derived variables and other parameters to enhance the understanding of cardiac mechanics in healthy individuals. The study was conducted in accordance with the Declaration of Helsinki (revised in 2013). Approval was obtained from the Institutional and Regional Human Biomedical Research Committee at the University of Szeged, Hungary (Registration Number: 71/2011; original approval 23 May 2011, latest approval date: 17 March 2025). All participants provided written informed consent prior to enrollment.

### 2.2. Two-Dimensional Doppler Echocardiography

Standard 2D echocardiography was performed in all subjects to measure left atrial and LV dimensions. The LV ejection fraction (EF) was calculated using the modified Simpson’s method. A Toshiba Artida^®^ cardiac ultrasound system (Toshiba Medical Systems, Tokyo, Japan), equipped with a broadband 1–5 MHz PST-30BT phased-array transducer, was used for all measurements. Doppler echocardiography was performed concurrently to exclude significant valvular stenosis or regurgitation and to quantify transmitral early (E) and late (A) diastolic flow velocities, as well as their ratio (E/A ratio) [18].

### 2.3. Three-Dimensional Speckle-Tracking Echocardiography

3DSTE studies were conducted in two phases [1,2,3,4,5]:

1. Data Acquisition

First, 3D echocardiographic datasets were acquired using the same Toshiba Artida^®^ system with a 3D-capable PST-25SX matrix-array transducer. After optimization of acquisition settings (e.g., magnitude, gain), 3D datasets were acquired from the apical window. With stable RR intervals on the ECG, subjects were asked to briefly hold their breath, while six subvolumes were acquired over six consecutive cardiac cycles. These individual datasets were then automatically stitched together by the software to create a full-volume dataset.

2. Offline Analysis

**Figure 1:**
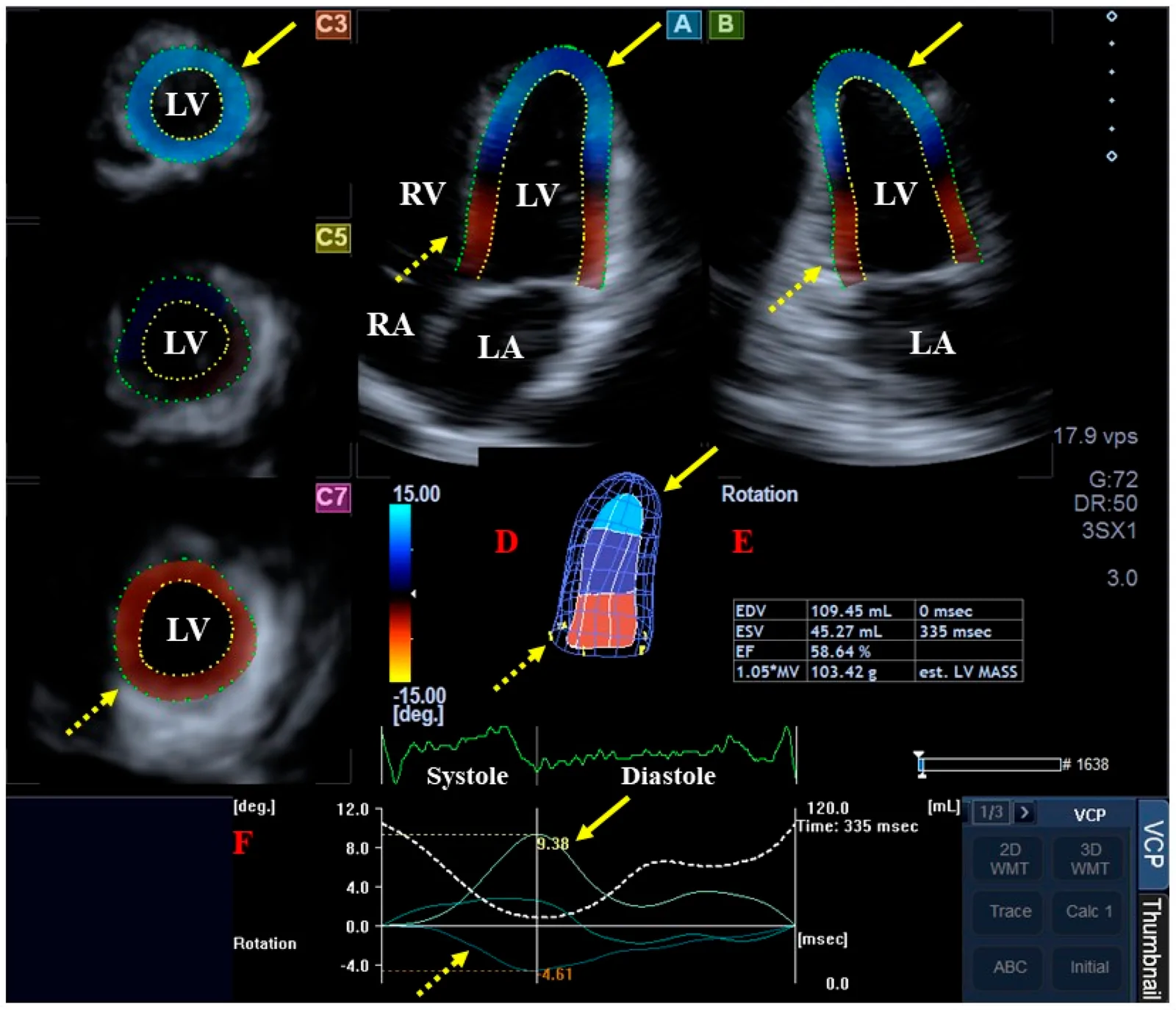
Assessment of left ventricular (LV) rotational parameters using three-dimensional (3D) speckle-tracking echocardiography. Representative apical four-chamber (**A**) and two-chamber (**B**) long-axis views are shown, along with short-axis views at the apical (**C3**), midventricular (**C5**), and basal (**C7**) levels. A virtual 3D reconstructed model of the LV (**D**) and calculated LV volumetric data (**E**) are also displayed. Apical (solid yellow arrow) and basal (dashed yellow arrow) LV regions and their time-LV rotational curves are presented, together with a curve representing temporal LV volume changes (dashed white line) throughout the cardiac cycle (**F**). Abbreviations. LV = left ventricle, LA = left atrium, RV = right ventricle, RA = right atrium.

The second stage involved the offline analysis of the acquired datasets at a later date, using vendor-provided dedicated software (3D Wall Motion Tracking version 2.7, Toshiba Medical Systems, Tokyo, Japan). LV strain parameters and AAPSE were measured using the previously acquired 3D echocardiographic datasets. The LV endocardial surface and the edges of the LV-mitral annulus were defined from apical two-chamber (AP2CH) and four-chamber (AP4CH) long-axis views, supplemented by three additional cross-sectional views at different LV levels. After optimizations, markers were placed to facilitate an automatic software-driven reconstruction of the LV endocardial surface and the creation of a 3D virtual cast of the LV following a sequential analysis. Using this LV model, the following LV rotational parameters were measured: basal and apical LV rotations, LV twist and LV twist time (Figure 1) [16,17].

**Figure 2:**
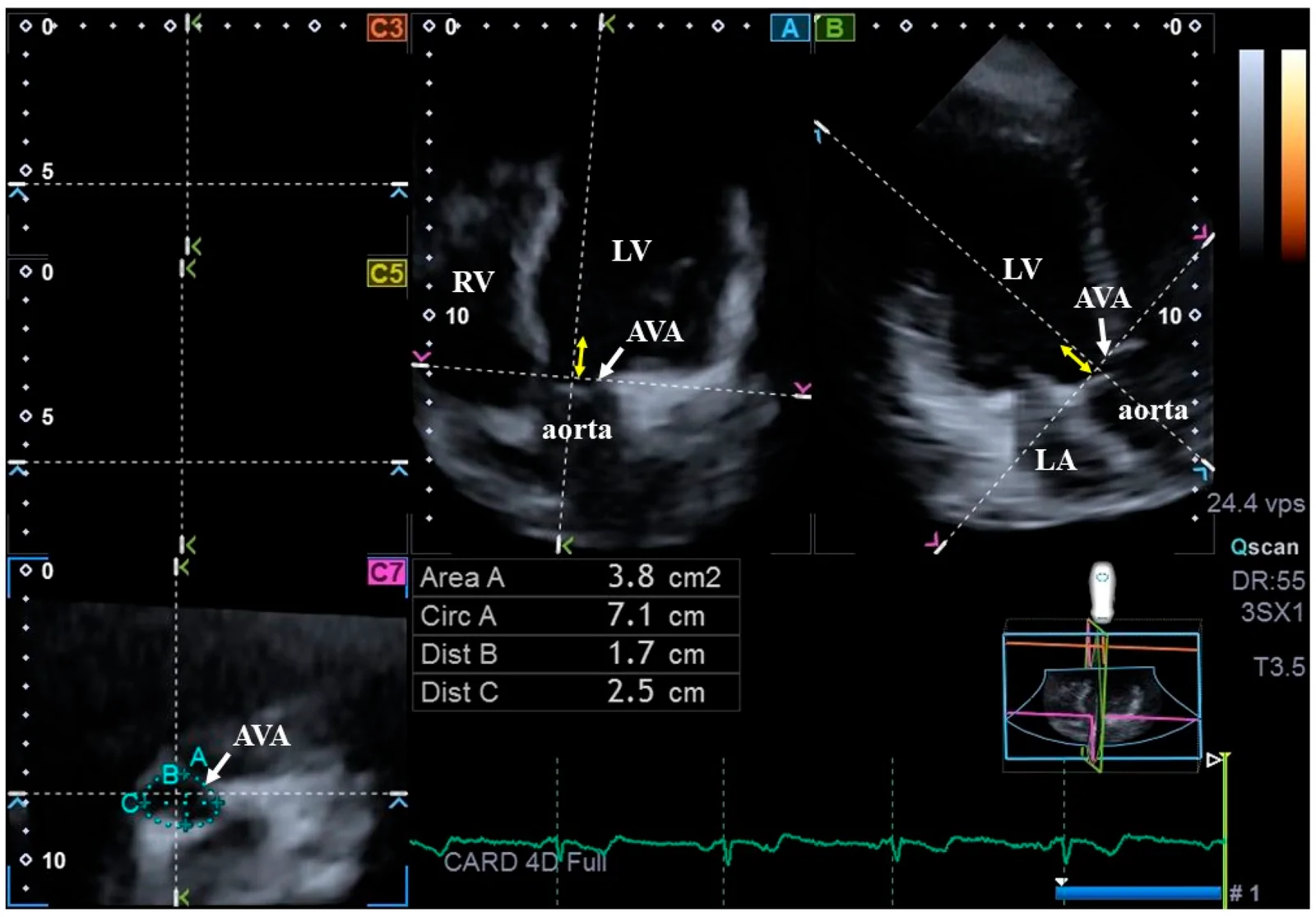
Detection of the aortic valve annular plane (see white arrow) in a three-dimensional echocardiographic database is shown, following optimization of the planes in both apical 2-chamber and 4-chamber long-axis views at the level of the aortic valve annular (AVA) plane during end-diastole (see ECG) and end-systole (**A**,**B**,**C7**). The displacement between these two time points is defined as the aortic annular plane systolic excursion (AAPSE, see yellow arrow).

To determine AVA position and measure AAPSE, AP4CH and AP2CH long-axis views were used to define optimal LV longitudinal planes. Visualization of the AV/aorta required tilting and optimization of these planes to be parallel to the centerline of the aortic root. The designated cross-sectional C7 view, used for alignment of the AVA, was positioned perpendicularly to the longitudinal plane. Care was taken during measurements to ensure that the C7 view remained truly perpendicular to the centerline and that measurements were not obtained within the sinus of Valsalva or the LV outflow tract. AAPSE was defined as the spatial displacement of the AVA measured between end-diastole and end-systole throughout the cardiac cycle (Figure 2) [15,17,19].

### 2.4. Statistical Analysis

Data are presented as mean ± standard deviation (SD) or as counts (percentages). Homogeneity of variances was assessed using Levene’s test, and normality was evaluated with the Shapiro–Wilk test. For normally distributed datasets, independent-samples *t*-test was used, whereas non-normally distributed data were analyzed using the Mann–Whitney-Wilcoxon test. Correlations were evaluated using Pearson’s correlation coefficients. Reproducibility of 3DSTE-derived measurements of LV rotational parameters and AAPSE was determined using mean ± 2SD differences for intraobserver and interobserver agreement, evaluated in 35 healthy individuals, along with intraclass correlation coefficients (ICCs). A two-sided *p*-value of less than 0.05 was considered statistically significant. All statistical analyses were performed using SPSS software (IBM SPSS Statistics for Windows, Version 29.0, IBM Corp., Armonk, NY, USA).

## 3. Results

### 3.1. Clinical Parameters

Clinical characteristics and conventional 2D echocardiographic data with Doppler analysis are summarized in Table 1. Importantly, none of the study participants exhibited significant valvular regurgitation (defined as grade 1 or greater) or clinically relevant stenosis affecting any cardiac valve.

### 3.2. Classification of Subjects

Healthy adults within the cohort were categorized based on mean ± SD values derived from 3DSTE analysis, specifically AAPSE (1.16 ± 0.30 cm), basal LV rotation (−4.06 ± 2.30 degrees) and apical LV rotation (9.34 ± 3.84 degrees). The following cut-off values, derived from mean ± SD ranges, were applied during the subsequent statistical analyses:

- for AAPSE: 0.86 cm and 1.46 cm.
- for basal LV rotation: −1.76 degrees and −6.36 degrees.
- for apical LV rotation: 5.50 degrees and 13.18 degrees.

Because the subgroup stratification was based on mean ± SD cut-off points derived from the current cohort, these analyses are inherently data-driven and must be considered strictly exploratory and hypothesis-generating.

### 3.3. LV Rotational Parameters in Different AAPSE Subgroups

A tendency toward increased basal LV rotation and decreased apical LV rotation was observed in healthy subjects with mean AAPSE, while LV twist remained preserved compared with those with lower- or higher-than-mean AAPSE values. Additionally, a progressive increase in LV twist time was observed in correlation with increasing AAPSE (Table 2).

### 3.4. AAPSE in Different LV Rotational Parameter Subgroups

A tendency toward increased AAPSE was observed in healthy subjects with mean basal LV rotation compared with those with lower- or higher-than-mean values. Furthermore, AAPSE was significantly increased in subjects with elevated apical LV rotation (Table 3).

### 3.5. Correlations

AAPSE did not correlate with any LV rotational parameters, including basal LV rotation (r = −0.008, *p* = 0.932), apical LV rotation (r = 0.065, *p* = 0.593), LV twist (r = 0.061, *p* = 0.632), and LV twist time (r = 0.181, *p* = 0.073).

### 3.6. Reproducibility of AAPSE and LV Rotational Parameters

Reproducibility parameters for AAPSE and LV rotational parameters are presented in Table 4.

## 4. Discussion

3D echocardiography has been validated as a robust method for measuring LV rotational mechanics [20,21,22] and AVA [23]. Moreover, 3DSTE enables not only the simultaneous evaluation of LV and AVA [17], but also the quantification of the spatial displacement of the AVA represented by AAPSE [15]. In addition, normal reference values of LV rotational parameters and AVA dimensions measured by 3DSTE have been well established [16,19]. In a recent study, no significant associations were found between LV rotational mechanics and AVA dimensions when assessed concurrently in healthy adults [17]. Although AAPSE measured by M-mode echocardiography was previously considered a characteristic of LV longitudinal function [13,14], the results of the MAGYAR-Healthy Study revealed that AAPSE, reflecting AVA motion, represents a more complex motion pattern, showing associations with 3DSTE-derived LV strain parameters [15]. However, in addition to the contraction–relaxation pattern, during the cardiac cycle [6], the LV exhibits a specific rotational mechanism, characterized by a clockwise basal LV rotation and a counterclockwise apical LV rotation [7,8,9,10,11]. Based on these considerations, the question arises as to whether and how AAPSE relates to LV rotational parameters measured simultaneously in healthy individuals, and whether these relationships differ across varying ranges of physiological values.

The present exploratory study has several implications. First, it has been demonstrated that the same 3D echocardiographic database can be used to quantify not only LV rotational parameters, but also the spatial displacement of the AVA as represented by AAPSE. Second, based on the results, a non-significant yet detectable increase in apical LV rotation was observed in subjects with elevated AAPSE. Interestingly, lower AAPSE values tended to be associated with decreased basal LV rotation and increased apical LV rotation. Third, AAPSE showed no association with the degree of basal LV rotation, but was significantly increased in the presence of elevated apical LV rotation. Characterizing this specific relationship between AAPSE and LV rotational mechanics in a healthy cohort provides a crucial, physiologically meaningful reference framework. Establishing the baseline characteristics of these functional parameters under normal conditions serves as a necessary prerequisite for clinical application. This framework is essential for future pathological studies, as it allows investigators to accurately detect the disruption of these normal physiological links, the onset of compensatory mechanisms, or the altered fiber dynamics characteristically seen in cardiovascular diseases. It would be worthwhile to investigate in future studies how these adaptive patterns manifest in specific pathologies or across different stages of heart failure. Fourth, despite these observable numerical trends, overall linear correlations between AAPSE and LV rotational parameters did not reach strict statistical significance, which underscores the exploratory nature of our findings. Fifth, these results should be interpreted in the context of LV strain parameters. A recently published study within the framework of the MAGYAR-Healthy Study confirmed that decreased AAPSE was associated with increased apical LV longitudinal strain (LS) [15], whereas basal LV-LS showed no association with AAPSE. In contrast, increased AAPSE was associated with elevated midventricular, apical, and global LV circumferential strain (CS), without significant relationships with LV radial strain (RS) or LV-LS. Although global LV-RS, LV-CS, and LV-LS did not reach statistical significance in their association with AAPSE, these parameters tended to increase when LV strain values deviated from the mean (either higher or lower) [15]. These findings highlight the complexity of LV function, in which impairment in one functional component may be counterbalanced by adaptive changes in another, thereby preserving overall pumping function. Another emerging modality described in the literature is the assessment of myocardial work by pressure-strain loop-derived indices. As complementary tools to strain and rotational mechanics (and AAPSE) in characterizing LV function, these parameters may underscore the need for further studies toward a deeper understanding of myocardial function [24].

## 5. Limitation Section

The following important limitations of the present study should be acknowledged:

- In the present study, the Toshiba Artida echo system was utilized in all cases. Given that 3DSTE measurements are known to differ across vendors, as supported by literature data, the generalizability of our reference values and relational findings is strictly limited. While the use of a single dedicated system for all examinations is a key strength of this study, the absolute values obtained might vary if equipment and analysis software from a different manufacturer were applied. These facts are crucial to consider when interpreting the findings and specific data.
- Although the study aimed to include apparently healthy individuals, it cannot be stated with absolute certainty that all participants were free of underlying conditions, as more extensive, supplementary diagnostic tests would have been required to definitively confirm their health status.
- The image quality of 3DSTE remains inferior to that of conventional 2D echocardiography. This limitation is primarily attributable to its reduced temporal and spatial resolution, constraints related to transducer size, and other technical factors, which may have affected the overall accuracy and applicability of the measurements [1,2,3,4,5].
- While 3DSTE-derived data acquisitions were performed between 2011 and 2017, AAPSE measurements were conducted in 2025 using previously stored 3D echocardiographic datasets. Although a significant period elapsed between data acquisition and analysis, the same equipment and software versions were utilized during the latter measurements to prevent any technical discrepancies or compounding variables. Admittedly, the Artida platform has since been updated, and newer generations of the analysis software have become available; however, these advancements represent future research opportunities leveraging the original datasets.
- The present study did not include comparative analysis with other established imaging techniques.
- The study design did not aim to directly compare the efficacy of the AVA and AAPSE measurements with those obtained using traditional M-mode echocardiography or other imaging methodologies.
- While 3DSTE is a versatile tool suitable for various other evaluations—such as comprehensive cardiac chamber quantifications and detailed examination of the atrioventricular valvular annuli—these potential analyses were determined to be beyond the defined scope and objectives of the current research [1,2,3,4,5].
- Despite the established close physiological relationship between AVA dimensions and the aorta, this interaction was not specifically investigated in the present study [12].
- Additionally, our study cohort represents a relatively young population (mean age of 35.1 years) with a predominance of male participants (62%). Given that LV myocardial rotational mechanics and AAPSE are highly age- and sex-dependent, the generalizability of our findings to older adults or cohorts with different sex distributions is constrained. Caution should be exercised when extrapolating these reference values, and further age- and sex-stratified analyses in broader populations are warranted.
- Finally, the stratification of our cohort using mean ± SD cut-off points derived from the same study population introduces potential circularity. Furthermore, numerous subgroup comparisons were performed without correction for multiple testing, which inflates the risk of type I error. Consequently, these subgroup analyses are exploratory by definition. Readers are cautioned against overinterpreting these subgroup trends, as they require independent validation in external, larger cohorts before any definitive clinical conclusions can be drawn.

## 6. Conclusions

There are no significant correlations between AAPSE and LV rotational parameters, but the trends observed in subgroup analyses could serve as hypothesis-generating observations for future studies.

## Figures and Tables

**Table 1:** Clinical and two-dimensional echocardiographic data.

Data	Measures
**Clinical data**	
*n*	110
Mean age (years)	35.1 ± 11.9
Males (%)	68 (62)
Systolic blood pressure (mmHg)	123 ± 3
Diastolic blood pressure (mmHg)	82 ± 2
Heart rate (bpm)	72 ± 3
Height (cm)	174.1 ± 12.3
Weight (kg)	73.2 ± 15.5
**Two-dimensional echocardiographic data**	
LA diameter (mm)	37.2 ± 3.6
LV end-diastolic diameter (mm)	48.0 ± 3.7
LV end-systolic diameter (mm)	32.1 ± 3.2
LV end-diastolic volume (mL)	107.0 ± 23.7
LV end-systolic volume (mL)	38.0 ± 9.3
Interventricular septum (mm)	9.2 ± 1.2
LV posterior wall (mm)	9.4 ± 1.5
LV ejection fraction (%)	64.6 ± 4.0
Early diastolic mitral inflow velocity—E (cm/s)	78.2 ± 16.4
Late diastolic mitral inflow velocity—A (cm/s)	59.1 ± 14.0

Abbreviations: LA = left atrial, LV = left ventricular.

**Table 2:** Aortic valve annular plane systolic excursion and left ventricular rotational parameters in different aortic valve annular plane systolic excursion subgroups.

	AAPSE ≤ 0.86 cm (n = 17)	0.86 cm < AAPSE A < 1.46 cm (n = 74)	AAPSE ≥ 1.46 cm (n = 17)
**AAPSE (mm)**	0.72 ± 0.08	1.15 ± 0.16 *	1.62 ± 0.16 *†
**basal LV rotation (°)**	−3.50 ± 2.12	−4.20 ± 2.31	−3.98 ± 2.69
**apical LV rotation (°)**	10.14 ± 2.69	8.79 ± 3.97	10.51 ± 3.98
**LV twist (°)**	13.65 ± 3.16	12.99 ± 4.52	14.57 ± 4.58
**LV twist time (ms)**	290 ± 82	346 ± 123	366 ± 143

* *p* < 0.05 vs. AAPSE ≤ 0.86 cm; † *p* < 0.05 vs. 0.86 cm ≤ AAPSE ≤ 1.46 cm. Abbreviations. AAPSE = aortic valve annular plane systolic excursion, LV = left ventricular.

**Table 3:** Aortic valve annular dimensions and left ventricular rotational parameters in different left ventricular rotational parameter groups.

	Basal LV Rotation ≤ −1.76 Degrees (n = 14)	−1.76 Degrees < Basal LV Rotation < −6.36 Degrees (n = 76)	Basal LV Rotation ≥ −6.36 Degrees (n = 20)	Apical LV Rotation ≤ 5.50 Degrees (n = 16)	5.50 Degrees < Apical LV Rotation < 13.15 Degrees (n = 76)	Apical LV Rotation ≥ 13.15 Degree (n = 18)
**AAPSE**	1.13 ± 0.29	1.18 ± 0.31	1.12 ± 0.28	1.19 ± 0.14	1.12 ± 0.30	1.31 ± 0.34 &
**basal LV rotation (°)**	−1.18 ± 0.61	−3.54 ± 1.11 *	−8.06 ± 1.18 *†	−4.02 ± 2.76	−4.10 ± 2.32	−3.80 ± 1.72
**apical LV rotation (°)**	8.99 ± 3.61	9.59 ± 3.94	8.67 ± 3.39	3.18 ± 1.64	9.09 ± 1.89 ‡	15.29 ± 1.37 ‡&
**LV twist (°)**	10.17 ± 3.94	13.13 ± 3.95 *	16.72 ± 3.70 *†	7.20 ± 3.40	13.19 ± 2.77 ‡	19.09 ± 2.41 ‡&
**LV twist time (ms)**	292 ± 123	349 ± 125	343 ± 88	331 ± 173	341 ± 106	349 ± 118

* *p* < 0.05 vs. basal LV rotation ≤ −1.76 degrees; † *p* < 0.05 vs. −1.76 degrees ≤ basal LV rotation ≤ −6.36 degrees; ‡ *p* < 0.05 vs. apical LV rotation ≤ 5.50 degrees; & *p* < 0.05 vs. 5.50 degrees ≤ apical LV rotation ≤ 13.15 degrees. Abbreviations. AAPSE = aortic valve annular plane systolic excursion, LV = left ventricular.

**Table 4:** Intra- and interobserver variability for aortic valve annular plane systolic excursion and left ventricular rotational parameters as assessed simultaneously by three-dimensional speckle-tracking echocardiography.

	Intraobserver Agreement		Interobserver Agreement	
	Mean ± 2SD Difference in Values Obtained by 2 Measurements of the Same Observer	ICC Between Measurements of the Same Observer	Mean ± 2SD Difference in Values Obtained by 2 Observers	ICC Between Independent Measurements of 2 Observers
**AAPSE (cm)**	−0.03 ± 0.14	0.91 (*p* < 0.01)	−0.02 ± 0.13	0.91 (*p* < 0.01)
**basal LV rotation (°)**	0.28 ± 0.13	0.80 (*p* < 0.01)	0.26 ± 0.18	0.81 (*p* < 0.01)
**apical LV rotation (°)**	0.04 ± 0.07	0.81 (*p* < 0.01)	0.58 ± 0.68	0.81 (*p* < 0.01)

Abbreviations. ICC = intraclass correlation coefficient, AAPSE = aortic valve annular plane systolic excursion, LV = left ventricular, SD = standard deviation.

## Data Availability

The original contributions presented in the study are included in the article; further inquiries can be directed to the corresponding authors.
